# Metacognitive Management of Attention in Online Learning

**DOI:** 10.3390/jintelligence12040046

**Published:** 2024-04-22

**Authors:** Matthew Jensen Hays, Scott Richard Kustes, Elizabeth Ligon Bjork

**Affiliations:** 1 Amplifire, Boulder, CO 80301, USA; skustes@amplifire.com; 2 Department of Psychology, University of California, Los Angeles, Los Angeles, CA 90095, USA; elbjork@psych.ucla.edu

**Keywords:** online learning, engagement, distraction, attention, distance learning, remote learning, technology in education, metacognition

## Abstract

Performance during training is a poor predictor of long-term retention. Worse yet, conditions of training that produce rapidly improving performance typically do not produce long-lasting, generalizable learning. As a result, learners and instructors alike can be misled into adopting training or educational experiences that are suboptimal for producing actual learning. Computer-based educational training platforms can counter this unfortunate tendency by providing only productive conditions of instruction—even if they are unintuitive (e.g., spacing instead of massing). The use of such platforms, however, introduces a different liability: being easy to interrupt. An assessment of this possible liability is needed given the enormous disruption to modern education brought about by COVID-19 and the subsequent widespread emergency adoption of computer-based remote instruction. The present study was therefore designed to (a) explore approaches for detecting interruptions that can be reasonably implemented by an instructor, (b) determine the frequency at which students are interrupted during a cognitive-science-based digital learning experience, and (c) establish the extent to which the pandemic and ensuing lockdowns affected students’ metacognitive ability to maintain engagement with their digital learning experiences. Outliers in time data were analyzed with increasing complexity and decreasing subjectivity to identify when learners were interrupted. Results indicated that only between 1.565% and 3.206% of online interactions show evidence of learner interruption. And although classroom learning was inarguably disrupted by the pandemic, learning in the present, evidence-based platform appeared to be immune.

## 1. Introduction

Many of the most effective conditions of learning are counterintuitive. As learners, we seem unable to accurately sense during the acquisition process whether we are optimally learning; that is, accomplishing learning that will be both long-lasting and generalizable. Or, in the theoretical framework put forth by Bjork and Bjork (1992, 2014), we are not able to feel whether a learning activity is generating broad, durable *storage strength* as opposed to narrow, ephemeral *retrieval strength*. Instead, what we as learners do readily perceive is difficulty, which we can mistakenly interpret as detrimental to our learning (e.g., Simon and Bjork 2001). As a result of these metacognitive misconceptions, we, as learners and also as instructors, tend to prefer and routinely choose inferior conditions of instruction over superior ones (e.g., Kornell and Son 2009).

An example of such a “desirable difficulty” (Bjork 1994) is the *spacing effect*: the phenomenon wherein long-term retention is dramatically better when the prior learning events have been separated by longer delays (i.e., *spaced*) than by shorter delays (i.e., *massed*). The spacing effect is perhaps the earliest demonstrated means for enhancing learning (Ebbinghaus 1885) and has proven to be remarkably robust (e.g., Cepeda et al. 2006). Spacing is beneficial across a wide range of test formats (see Crowder 1976 for an extensive review) and across many types of to-be-learned materials, from meaningless keystroke patterns (e.g., Simon and Bjork 2001) to learning a foreign language (e.g., Bahrick et al. 1993). And spacing appears to be beneficial for every conceivable type of learner, including adults (e.g., Baddeley and Longman 1978), children (e.g., Toppino 1993), and even sea slugs (Carew et al. 1972). Given the robustness of this phenomenon, it seems that it should have already been incorporated into nearly all educational programs and settings, and that students should gravitate to it by default. 

Unfortunately, however, learners’ misinterpretation of difficulty as detrimental leads them astray. For example, given the choice between more versus less spacing, students choose less (Wissman et al. 2012). Given the option to mass together or space out learning events, students choose massing (Tauber et al. 2012). Our misguided intuition even overpowers our own experience; learners who have just demonstrated superior retention in a spaced-practice condition still believe that massed practice is better (e.g., Kornell and Bjork 2008), even when they have been strongly warned against trusting such beliefs (e.g., Yan et al. 2016). This pattern of poor learner-managed learning extends beyond spacing-versus-massing to many other well-established cognitive phenomena (Bjork 1994). 

### 1.1. Technological Advancement

Fortunately, advances in technology coupled with informed educational design can sidestep such metacognitive errors and their resulting detrimental choices. That is, by guiding learners’ activities via a properly informed computer-constructed program, individuals can learn effectively without needing to become metacognitively sophisticated regarding how best to learn. For example, spacing can be enforced algorithmically, as opposed to its use being left to the whim of the student. 

Digital education has other advantages, as well. Learners can adjust the pace of online classwork to fit their unique needs (e.g., Sølvberg and Rismark 2012), and students often benefit academically when they have a greater sense of agency in their education (Sorgenfrei and Smolnik 2016). More advanced computerized learning systems can tailor each instructional event to a learner’s real-time needs, both actual (e.g., Hays et al. 2019) and perceived (Henderson et al. 2017). 

These benefits explain the (pre-pandemic) proliferation of digital educational tools. Many well-established universities now feature course companions (online adjuncts to in-person classes). Students increasingly use eBooks rather than hauling backpacks full of course readers across campus. Some traditional universities have begun to offer online degree programs (e.g., the MS in Computer Science from Georgia Tech; McKenzie 2018). Other schools are hosted entirely online; over 50,000 students are enrolled online at Western Governors University, Southern New Hampshire University, the University of Phoenix, and others (Bushra 2022).

### 1.2. Technological Vulnerability

Despite their advantages and increasing popularity, however, computer-guided learning platforms have a critical vulnerability. No matter how rigorously an online platform is designed to enforce productive, counterintuitive conditions of instruction, students still have complete control over the most basic metacognitive decision of all: how attentive they will be to the lesson. And although many teachers fall prey to the same misconceptions as students (e.g., preferring massing to spacing in their lessons or lectures), more biology is learned from an hour attending a poorly designed biology lecture than from an hour of distracted scrolling through pictures of cats. (For a comprehensive review of self-regulated learning in digital environments, see Wong et al. 2019). 

In a classroom, the physical and social aspects of the learning environment are designed to reduce the number of distractions and learners’ propensity to succumb to them. Seats generally face the instructor. Everyone in the room at class time is ostensibly intending to participate in the same learning experience. Chatting with other students is typically discouraged. The use of personal mobile devices is generally prohibited. Moreover, it is considered rude to ignore the lesson in a classroom and even more rude to disrupt the education of others. These environmental and social guardrails usually encourage students to stay at least somewhat engaged (e.g., Forrin et al. 2021; Kalsi et al. 2023), and instructors can often detect and redirect distracted learners. 

### 1.3. Interruptions during Digital Learning and Their Consequences

In contrast, online education lacks many of these attention-directing cues (e.g., Sitzmann et al. 2010; Terras and Ramsay 2012). It is far easier for a learner to be distracted from a screen than from classroom-led instruction (e.g., Aivaz and Teodorescu 2022; Conrad et al. 2022). Further, online education permits far more sources of interruption, including phone calls, error messages, text-message alerts, dropped connections, social media notifications, and more (Wang 2022). 

When these distracting events pull students’ attention away from instructional content, their retention of the material suffers (e.g., Blasiman et al. 2018; Sitzmann et al. 2010). Being interrupted also incurs an opportunity cost; during the period of disengagement, the student could have instead been spending their time on productive learning activities. Kraft and Monti-Nussbaum (2021) found that students in classrooms lose the equivalent of 10–20 days of school per year to interruptions; the amount for online learners is surely greater. Resuming a task after interruption is difficult (Federman 2019) and the resumption process takes time (Dontre 2021). When students are finally able to resume the task, their post-interruption learning is slowed (e.g., van de Poll and Sörqvist 2016), compounding the opportunity cost. In summary, disruption during learning is undesirable on several fronts. 

### 1.4. Interruptions, Online Learning, and COVID-19

On 11 March 2020, the World Health Organization declared COVID-19 to be a pandemic (WHO 2020). In response, most governments and educational institutions implemented measures to “stop the spread” (i.e., reduce the number of new infections; Bentley and Cahill 2021) and “flatten the curve” (i.e., reduce the risk of overloading healthcare systems; CDC 2007). One such measure was “lockdowns”, which restricted travel, movement, and in-person interaction. It is impossible to overstate the impact of these measures on everyday life. Lockdowns even *changed how birds sing* (Derryberry et al. 2020).

Within a few days, 94% of students’ in-person education either ended or was abruptly replaced with online substitutes (UNESCO 2021). Learning suffered; Hodges et al. (2020) clearly differentiate “emergency remote teaching” from “well-planned online learning experiences”. But a substantial contributor to impaired learning was the stress caused by COVID-19 and lockdowns. Students worldwide were overwhelmed by concerns about the virus (e.g., fear for one’s own health or the health of family members) or the upheaval caused by lockdowns (e.g., isolation from classmates and friends). Almost all students in a study by Son et al. (2020) reported disrupted sleep. Anxiety rates tripled (Davenport et al. 2020; Santabárbara et al. 2021). Children’s Hospital of Colorado declared its first “state of emergency” in 117 years of operation not due to the virus but because suicide became the leading cause of children’s death (CHCO 2021).

COVID-19 and lockdowns also exacerbated other stressors. There were substantial increases in alcoholism (White et al. 2022), domestic abuse (Piquero et al. 2021), and the “shadow pandemic” of violence against women (e.g., Ravindran and Shah 2023). Prescription medications became unavailable (Bookwalter 2021). Millions of people lost their primary source of income (Cannon et al. 2021). Food insecurity and starvation were widespread (Kakaei et al. 2022). Vulnerable populations suffered disproportionately (Li et al. 2023). 

Unsurprisingly, stressors of this magnitude can make it difficult to pay attention (Banks and Boals 2017; Boals and Banks 2020). Son et al. (2020) found that 89% of students reported difficulty concentrating as a result of COVID-19 and lockdowns. Other self-reported measures of attention to instruction also decreased (Copeland et al. 2021), and students indicated increased anxiety, mind wandering, and external distractions (Hicks et al. 2023). 

Under these extraordinary circumstances, what happened to students’ ability to resist interruptions while learning online?

## 2. The Present Study

The present study had three purposes. The first was to explore straightforward methodological approaches (i.e., that an instructor could readily implement) for identifying interruptions in a digital learning experience. The second was to use these approaches to describe the frequency at which students are interrupted during a digital learning experience specifically designed to incorporate features shown to enhance long-term retention. 

The third purpose of the present study was to examine whether the frequency of any such interruptions was increased owing to the pandemic and ensuing lockdowns. Our hypothesis was that the disruption from COVID-19 and lockdowns extended to all aspects of students’ education, including adaptive digital learning environments. 

In the United States (from where most of the data we analyzed had been collected), lockdowns were generally in place from mid-March 2020 through May 2020 (Pallay 2021). We defined *pre-COVID* as January 2019 to February 2020, inclusive; *peak COVID* as April and May 2020; and *post-peak* as June 2020 to December 2022, inclusive. Because March 2020 was the transition period, it was excluded from these time periods. 

Our hypothesis: We expected to see an increase in the frequency of interruptions from pre-COVID to peak COVID. In large data sets (as in the present study), tests for statistical significance are not informative (Altman 1980). In such cases, effect size is far more useful for indicating whether a difference is meaningful. Savage et al. (2020) found medium-sized effects of the pandemic on mental well-being and perceived stress, which can negatively impact attention (Banks and Boals 2017). Similarly, Davenport et al. (2020) found moderate and large increases in depression and anxiety, respectively. Maldonado and De Witte (2022) found small (*d* = 0.17 to 0.19)^1^ but reliable effects on learning, as did Oostdam et al. (2023). Hicks et al. (2023) found large decreases in self-reported ability to focus and large increases in self-reported mind wandering and external distractions. Based on this evidence, we set our smallest effect size of interest (SESOI; Lakens 2014) to be a medium effect (equivalent to *d* = 0.5). We expected the post-peak interruption rate to fall but to still be above the pre-COVID rate; for that difference, our SESOI was small (equivalent to *d* = 0.3).

An alternative hypothesis would be that disruptions due to COVID-19 would be mostly confined to learning experiences that were directly disrupted (e.g., a college course that underwent an emergency transition from in-person to remote). Because the learning platform in the present investigation had been, from the start (and long before the pandemic), exclusively intended for online use, students’ experiences in it may have been inoculated against such a disruption. In that case, we would expect to see relatively consistent interruption rates across the three periods. 

To control for any potential effects of seasonality, we also compared the peak COVID period (April and May of 2020) to those same months in 2019, 2021, and 2022. Our SESOI was medium for the comparison to 2019 and small for the comparisons to 2021 and 2022. 

### 2.1. Methodological Note

In a typical laboratory experiment, participants agree to complete a series of tasks in return for research participation credit or remuneration. They are explicitly aware that the details of their performance will be scrutinized, and the shared presumption is that the participant will focus on the task until it is complete. This approach has been used in numerous evaluations of attention, distraction, and interruption (e.g., Marone et al. 2018). 

However, because the experimental context can artificially inflate measures of engagement and motivation (e.g., Feil et al. 2002), it could meaningfully obscure our efforts to delineate the frequency of interruption during online learning. Research participants sometimes change their behavior to fit experimenters’ hypotheses (e.g., Nichols and Maner 2008) or to fit their perception of experimenters’ expectations (Weber and Cook 1972).

Instead of estimating interruption frequency in an experimental context, other researchers have elected to rely on self-report (e.g., Becker et al. 2013). By various methods, learners are surveyed and asked to estimate how often various interruptions disconnected them from their coursework (e.g., Schmidt 2020). Unfortunately, learners’ memories are inaccurate (e.g., Deslauriers et al. 2019; Yan et al. 2016) and similar demand characteristics could affect their responses. 

We therefore elected in the present research to examine only data passively collected from learners as they completed online educational tasks associated with their university coursework. 

### 2.2. Data Collection Platform

The data were collected by an adaptive, online educational platform that employs robust—although often counterintuitive—principles of cognitive science to make the material “stick” (Brown et al. 2014). These include spacing, priming by pre-testing (Kornell et al. 2009; Soderstrom and Bjork 2023), requiring simultaneous confidence indication and answer selection (Soderstrom et al. 2015), delaying corrective feedback to the extent it is beneficial (Butler and Roediger 2008; Swinnen et al. 1990; for a review, see Kulik and Kulik 1988), repeatedly testing material until it is mastered (e.g., Karpicke and Roediger 2007), interleaving related topics (e.g., Gulacar et al. 2022; Kornell and Bjork 2008), selectively presenting multimedia (Mayer and Fiorella 2014), and more. (For an in-depth system description, please see Hays et al. 2019).

The software platform presented learners with interactive educational modules that functioned as companion exercises for digital or physical textbooks. Each module roughly corresponded to a textbook chapter and was composed of around two dozen items; each item comprised a question, its correct and incorrect responses, and its corresponding explanatory feedback designed to correct misunderstandings. Most questions were in multiple-choice format, but small percentages were matching (i.e., drag-and-drop) or multi-correct (i.e., select all that apply). 

The online-learning platform algorithmically determined whether, after the learner responded to a question, the corresponding corrective feedback needed to be presented (Pashler et al. 2005). The platform also determined whether the learner would need to encounter the question again or if the underlying concept had reached an algorithmically determined mastery state. An item was only considered complete when it had been mastered, and a module was only considered complete when all its items had been mastered; learners could not simply click through in order to end the learning experience. As a result, one learner might encounter a question once and never see its feedback, while another learner might encounter a question and its corrective feedback several times. We evaluated whether each attempt to answer a question was interrupted. We also evaluated whether each encounter with corrective feedback was interrupted. These attempts and encounters are collectively referred to as *interactions*.

### 2.3. Study Overview

Most digital educational platforms do not afford direct observation of the learners. As a result, interruptions must be inferred from the available data. One of the few clean variables available to us (and to future investigators working with similar web-based systems) is interaction duration. 

Given that an interruption is an event that disengages focus and temporarily stops progress on a task (Sitzmann et al. 2010), we used excessive time spent during an interaction as a proxy for interruption and turned to outlier-detection methods to define “excessive”. Although outliers are usually identified with the goal of removing them (e.g., McClelland 2014), in this case they represent precisely the duration-of-interaction aberrations we aimed to quantify. In other words, outlier-detection approaches should indicate when someone is doing something other than learning while “learning”. 

In Study 1, we used a straightforward mean-and-standard-deviation-based approach to defining outliers—and, thus, interruptions. In Study 2, we adjusted our analysis to factor in each learner’s relative interaction speed, thereby accounting for students’ individual differences. In Study 3, we restricted our analysis to avoid a potential confounding variable: the patterns of textbook edition publishing and distribution over time. In Study 4, we used a more robust approach to outlier detection. This approach allowed us to avoid using popular but ultimately arbitrary cutoffs for defining an outlier and instead allowed the data to tell us when an interaction lasted long enough for us to believe that the learner had been interrupted. In Study 5, we used 30-plus-minute software-inactivity timeouts as unequivocal indicators of long interruptions.

## 3. Study 1

### 3.1. Method

#### 3.1.1. Research Population

The data used in this study were collected from 1,409,480 university students across the world as they completed modules in the online-learning platform. Most of these students were enrolled at traditional universities where their assignments were online components of in-person courses. Other students were enrolled at hybrid or online-only universities. (Because the data were anonymized, there was no way to distinguish the two). 

#### 3.1.2. Materials

The instructional modules covered 17 different college-level topics, such as physics, accounting, biology, food-service safety regulations, and psychology. There were 8377 authored modules from a total of 177 textbooks. Each module comprised an average of 23.20 authored items. Learners took an average of 24.27 min to complete each module. Most learners encountered content from one or two textbooks (*M* = 1.08); no learner encountered all the material. 

All data were from modules started and completed between 2019 and 2022, inclusive. The total data set comprised 20,390,650 module completions and 1,018,463,722 interactions (806,854,539 responses to questions and 211,609,183 reviews of corrective feedback). Completing a module took an average of 49.95 interactions.

### 3.2. Results and Discussion

For each authored item, means and standard deviations (SDs) were separately calculated for the duration of learners’ (a) initial attempts, (b) subsequent attempts, (c) initial reviews of corrective feedback, and (d) subsequent reviews of corrective feedback. For each of these interaction types for each item, interruptions were defined as interactions lasting longer than the mean + 2 SD of those interactions for that item—a common threshold for defining an outlier in the social sciences (Miller 1991). 

**Figure 1:**
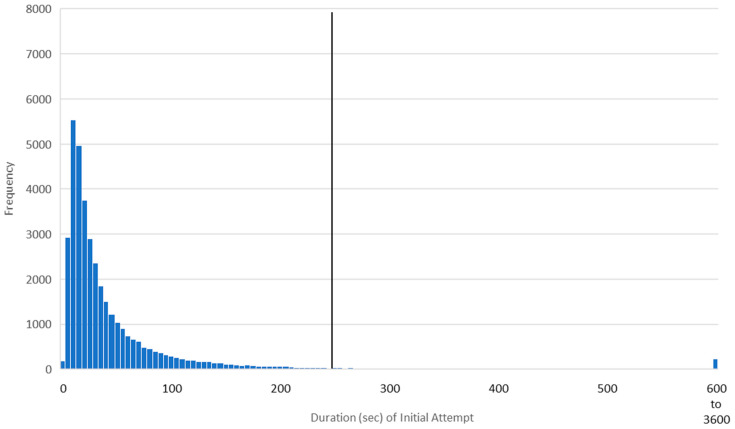
Distribution of durations of initial answer attempts for a single question. The mean + 2 SD interruption threshold (252.52 s) is indicated by a vertical line. Interactions longer than 600 s were condensed for visual clarity.

Figure 1 presents the distribution of the durations of the initial attempts for a single question encountered by 37,521 learners. As can be seen, the data were positively skewed (median = 28.00 s, mean = 54.60 s, SD = 98.96 s). It is also visually apparent that the threshold for interruption (mean + 2 SD = 252.52) is far past the bulk of the learners’ responses. 

**Figure 2:**
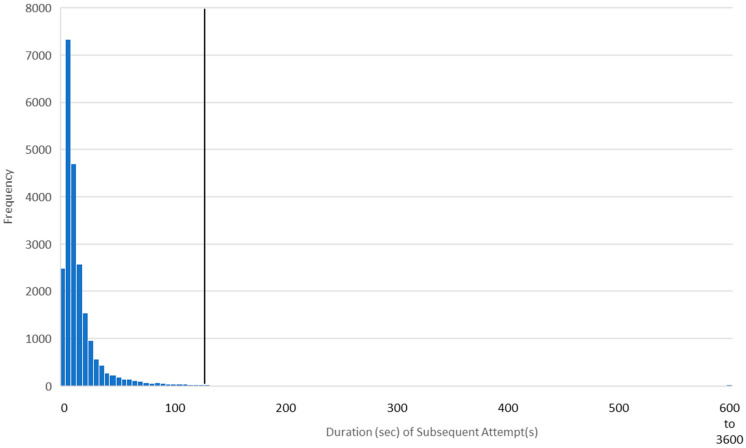
Distribution of durations of subsequent answer attempts for a single question. The mean + 2 SD interruption threshold (113.71 s) is identified by a vertical line. Interactions longer than 600 s were condensed for visual clarity.

Figure 2 presents the distribution of the durations of subsequent (i.e., non-initial) attempts to answer the same question as was depicted in Figure 1. The platform determined that some learners did not need to see the question more than once; thus, only 22,521 interactions from 10,662 learners were in the subsequent-attempt distribution. As with the initial attempt, the data were positively skewed (median = 12.00 s, mean = 20.74 s, SD = 46.48 s). Again, the threshold for interruption (113.71 s) is well beyond the duration of most learners’ responses. (As can be seen, subsequent-attempt interactions were much quicker than initial-attempt interactions, which is what led us to calculate outliers separately for each of the four interaction types). 

Together, this pattern of results suggests that the mean + 2 SD approach effectively identifies when a learner has spent an anomalous amount of time on a learning activity and can thus be interpreted as indicating that the learner was at least temporarily disengaged from that learning activity (i.e., interrupted). 

#### 3.2.1. Interruption Frequency

To measure the aggregate interruption rate across all learners and content, we divided the total number of interrupted interactions (i.e., interruptions) by the total number of interactions, combined across interaction types. The result was an overall mean interruption rate of 2.017%. This rate translates to approximately one interruption per module. This finding appears to be consistent with evidence that the software platform produces durable, long-term knowledge gains and skill improvements (Hays et al. 2019; Lowery et al. 2022). It should be noted that, although interruptions were rare, they were relatively lengthy; interruptions accounted for an average of 7.21 min (29.70%) of learners’ logged time in a module. 

#### 3.2.2. Interruptions and COVID-19

**Figure 3:**
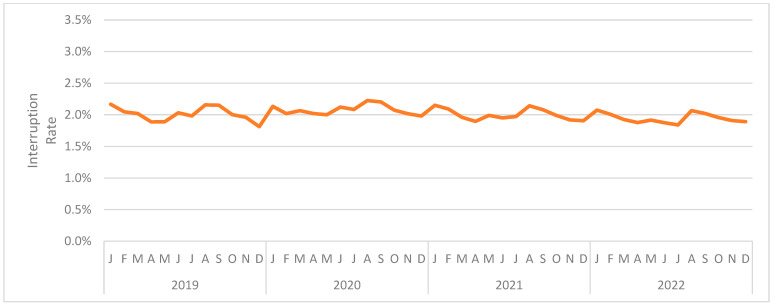
The rate of interruptions by month.

Figure 3 presents the interruption rate (combined across interaction types) by month. The (unweighted) mean monthly interruption rate was 2.009%^2^. The month with the highest mean interruption rate was August 2020 at 2.225%. The month with the lowest mean interruption rate was December 2019 at 1.813%. As can be seen, the interruption rate was relatively consistent across the period under analysis. 

Table 1 presents the interruption counts, interaction counts, and interruption rates for the time periods corresponding to our hypothesis. As can be seen, the numeric differences in the interruption rates appear trivial.

Hypothesis tests were performed using R statistical software (v4.3.0; R Core Team 2023). We used the prop.test() command to conduct a test of equal proportions (Newcombe 1998), which indicated that pre-COVID, peak COVID, and post-peak interruption rates were statistically significantly different: χ^2^(2, *N* = 995,687,507^3^) = 107.16, *p* < .000000000000000022. However, as mentioned above, this statistical significance is driven by the sample size; in a data set this large, any numeric difference whatsoever would have been statistically significant (Altman 1980). 

We therefore used effect size to compare the pairs of time periods in our hypothesis. To compare two proportions $p_{1}$ and $p_{2}$, Cohen (1988) gives the following formula for effect size *h*:

$$h=2arcsin\left(\sqrt{p_{1}}\right)-2arcsin\left(\sqrt{p_{2}}\right)$$

According to Cohen (1988), an *h* of 0.2 represents a small effect and an *h* of 0.5 represents a medium effect. Comparing pre-COVID to peak COVID yielded an effect size of *h* = 0.00069. Comparing pre-COVID to post-peak yielded an effect size of *h* = 0.00071. Comparing peak COVID to post-peak yielded an effect size of *h* = 0.00003. Despite being statistically significant, these differences are practically negligible. 

Table 2 presents the interruption counts, interaction counts, and interruption rates for the seasonality-controlled time periods in our hypotheses. As in Table 1, the numeric differences in interruption rates appear trivial. 

A test of equal proportions indicated that interruption rates in the four time periods were statistically significantly different: χ^2^(3, *N* = 152,778,376) = 2186.2, *p* ≈ 0. Comparing 2020 to 2019 yielded an effect size of *h* = 0.00904. Comparing 2020 to 2021 yielded an effect size of *h* = 0.00647. Comparing 2020 to 2022 yielded an effect size of *h* = 0.00900. In all cases, to quote Lakens (2017), “the true effect is close enough to zero for our practical purposes” (p. 355).

Contrary to our hypothesis, the data appear to indicate that COVID-19 did not substantially disrupt learning in the online educational platform used in the present evaluation. This finding is consistent with the alternative hypothesis that, despite causing profound disruptions to education and life more broadly, the pandemic and lockdowns did not meaningfully alter the rate of interruptions in a platform that was not part of the emergency transition from classroom to computer. (We also looked at the daily interruption rate in March 2020 to see if its exclusion was inappropriate, but the daily rate never exceeded 2.8%. For comparison, the interruption rate on Christmas 2020 was nearly 4%).

#### 3.2.3. Limitations

In any classification system, false positives are balanced against false negatives (e.g., Bentley et al. 2012). Based on the appearance of the distributions in Figure 1 and Figure 2, our threshold appears appropriate. But—compared to a more sophisticated approach—a single, fixed threshold may allow too many false positives (i.e., normal interactions labeled as interruptions) while also allowing too many false negatives (i.e., interrupted interactions labeled as normal).

Furthermore, although the mean + 2 SD approach is straightforward, it fails to take into account individual differences among learners. Slower learners may have the same true interruption rate as faster learners, but because their interactions generally take longer, they may have been more likely to be flagged as interrupted. Similarly, fast learners may have been less likely to be flagged as interrupted. 

Study 2 was designed to address these two limitations.

## 4. Study 2 

For Study 2, we created a *speed factor* by which the mean + 2 SD threshold could be adjusted in order to take into account learners’ individual differences. A learner’s speed factor for a particular interaction type (e.g., initial question attempt) was calculated as the ratio between the total duration for that interaction type *for that learner* divided by the total mean duration for that interaction type *for all learners*, restricted to the items that the learner encountered. 

To illustrate: suppose a particular learner’s initial responses to Items 1, 2, and 3 took 20, 20, and 30 s, respectively. Suppose the mean initial response durations for those three items across all learners were 20, 40, and 60 s, respectively. The initial-attempt speed factor for this particular learner would be

$$\frac{20+20+30}{20+40+60}=0.58333$$

For this particular learner, the interruption threshold for the initial attempt on the example item shown in Figure 1 would be (0.58333 × 252.52) = 147.30 s instead of 252.52. That is, if this hypothetical learner had responded in 200 s in Study 1, their response would have been categorized as normal; in Study 2, it would have been categorized as interrupted. For slower learners (i.e., learners with speed factor values above 1.0), interactions could move in the opposite direction, from categorized as interrupted to categorized as normal. 

**Figure 4:**
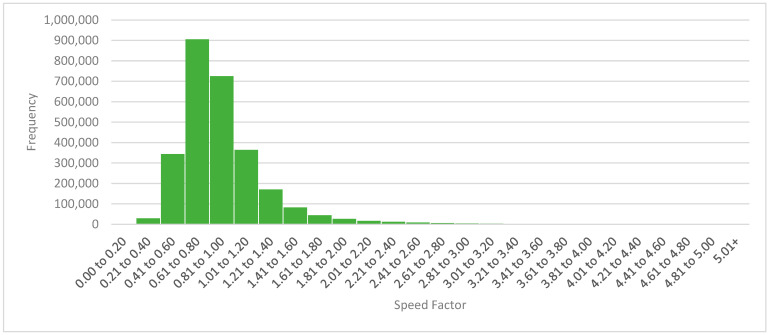
The distribution of initial-attempt speed factor for all learners.

Figure 4 presents the distribution of all learners’ speed factors for initial attempts. 

### 4.1. Method

The research population and materials were the same as in Study 1. 

### 4.2. Results and Discussion

For each authored item, the mean + 2 SD thresholds were carried over from Study 1 for that item’s (a) initial attempts, (b) subsequent attempts, (c) initial reviews of corrective feedback, and (d) subsequent reviews of corrective feedback. Within each of these interaction types for each item, interruptions were defined as interactions lasting longer than the mean + 2 SD of those interactions for that item. The item’s threshold for each type of interaction, for each learner, was then adjusted by that learner’s speed factor for that interaction type. 

**Figure 5:**
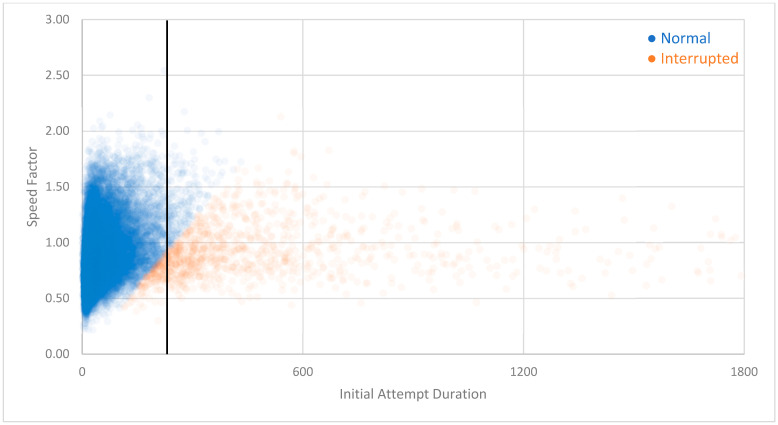
Scatterplot depicting the number of interactions at each combination of learner speed factor and initial attempt duration for the item depicted in Figure 1. Marker transparency is set to 95%; darker colors indicate more data at a particular intersection of speed and factor interaction duration. The black vertical line indicates the speed-factor-agnostic interruption threshold from Study 1 (252.44 s), which is included as a point of reference. Orange interactions to its left have gone from “normal” to “interrupted”, and blue interactions to its right have gone from “interrupted” to “normal”.

Figure 5 presents a scatterplot of learners’ initial-attempt durations versus their initial-attempt speed factors^4^ for the item shown in Figure 1. The speed-factor-agnostic threshold from Study 1 is included as a point of reference. As can be seen (and as was expected), some interactions previously categorized as normal became categorized as interrupted. A smaller number of interactions previously categorized as interrupted became normal. 

This pattern of results suggests that incorporating the speed factor improves the interruption-detection approach introduced in Study 1. It accommodates slower learners and reduces false positives. Simultaneously, using the speed factor reduces false negatives from faster learners with briefer interruptions.

#### 4.2.1. Interruption Frequency

As in Study 1, we measured the aggregate interruption rate across all learners and content by dividing the total number of interruptions by the total number of interactions, combined across interaction types. The result was an overall mean interruption rate of 2.855%. This rate translates to approximately 1.43 interruptions per module. 

The higher interruption rate in Study 2 versus Study 1 suggests that incorporating the speed factor eliminates more false negatives than false positives. This makes sense, given that there were more data available for reclassification below the threshold than above it. 

#### 4.2.2. Interruptions and COVID-19

**Figure 6:**
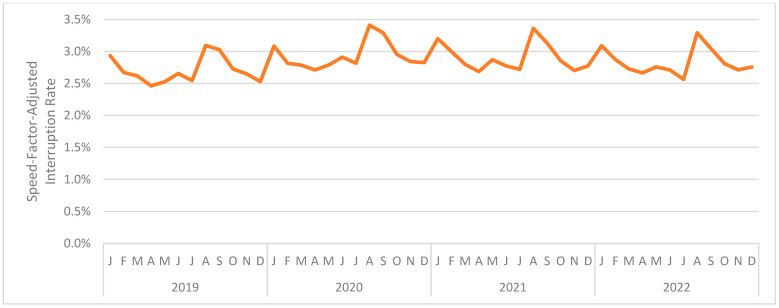
The monthly interruption rate for Study 2.

Figure 6 presents the interruption rate (combined across interaction types) by month. The (unweighted) mean monthly interruption rate was 2.845%. The month with the highest mean interruption rate was August 2020 at 3.412%. The month with the lowest mean interruption rate was April 2019 at 2.462%. As can be seen, and as was the case in Study 1, the interruption rate was relatively consistent across the period under analysis. 

Table 3 presents the interruption counts, interaction counts, and interruption rates for the time periods corresponding to our hypothesis. As can be seen, and as was the case in Study 1, the numeric differences in the interruption rates appear trivial. 

A test of equal proportions indicated that pre-COVID, peak COVID, and post-peak interruption rates were statistically significantly different: χ^2^(2, *N* = 995,687,507) = 23,881, *p* ≈ 0. Again, this statistical significance is driven by the sample size, so we used effect size to compare the pairs of time periods in our hypothesis. Comparing pre-COVID to peak COVID yielded an effect size of *h* = 0.00063. Comparing pre-COVID to post-peak yielded an effect size of *h* = −0.01027. Comparing peak COVID to post-peak yielded an effect size of *h* = −0.01090. As in Study 1, these differences are negligible. 

Table 4 presents the interruption counts, interaction counts, and interruption rates for the seasonality-controlled time periods in our hypotheses. As in Table 3, the numeric differences in interruption rates appear trivial. 

A test of equal proportions again indicated that interruption rates in the four time periods were statistically significantly different: χ^2^(3, *N* = 152,778,376) = 6783, *p* ≈ 0. Comparing 2020 to 2019 yielded an effect size of *h* = 0.01585. Comparing 2020 to 2021 yielded an effect size of *h* = −0.00026. Comparing 2020 to 2022 yielded an effect size of *h* = 0.00248. The true effect of lockdowns on interruptions in the present study is effectively zero. 

These findings are consistent with Study 1 and inconsistent with our hypothesis; COVID-19 did not substantially disrupt learning in the online educational platform used in the present evaluation. Indeed, looking back at Figure 6, the beginning of each semester (January; August/September) seems to have been more disruptive than COVID-19 and lockdowns. 

## 5. Study 3

The relative nature of the mean + 2 SD outlier threshold yields a potential weakness in our analysis of the impact of the pandemic. The content used in university classrooms changes over time as new editions of textbooks are released and make their way into campus bookstores. In Studies 1 and 2, outliers were determined on an item-by-item basis, but each item is contained within a particular edition of a textbook. These two factors together could conceivably create the illusion that there was no effect of lockdowns on interruptions when such an effect was actually present. For example, suppose 2020 had far more real-world or “true” interruptions but also that content used in 2020 was primarily only used in 2020. The result would be that more frequent true interruptions increased the mean and SD for that 2020-specific content, thereby inflating the threshold and making the flagged interruption rate in 2020 still appear similar to the rate in the other years (Jones 2019). 

In Study 3, we restricted our analyses to learners’ interactions with modules from a single textbook. This textbook had approximately the same number of interactions in each year under analysis, which eliminated differential usage over time as a potential confounding variable. This restriction ensures that reported effects do not emerge from Simpson’s (1951) paradox, where different subpopulation sizes cause local trends and global trends to conflict.

### 5.1. Method 

#### 5.1.1. Research Population

The data used in the present study were collected from 72,380 university students across the world as they completed modules in the online-learning platform as part of their coursework. This is a subset of the data set used in Studies 1 and 2. 

#### 5.1.2. Materials

The instructional modules came from a single anatomy and physiology textbook. This textbook’s online component contained 89 authored modules, each comprising an average of 26.16 authored items. Learners took an average of 21.57 min to complete each module. 

All data were from modules started and completed between 2019 and 2022, inclusive. The total data set comprised 1,848,708 module completions and 98,893,955 interactions. Completing a module took an average of 53.49 interactions. There were approximately the same number of interactions in each year: 25,597,264 in 2019; 25,078,524 in 2020; 23,716,017 in 2021; and 24,502,150 in 2022. 

### 5.2. Results and Discussion

For each authored item, the mean + 2 SD threshold was carried over from Study 1 for that item’s (a) initial attempts, (b) subsequent attempts, (c) initial reviews of corrective feedback, and (d) subsequent reviews of corrective feedback. Interruptions were defined as they were in Study 2, although the speed factor for Study 3 was calculated only based on users’ interactions with the anatomy and physiology textbook to which we restricted this analysis. (The distribution of the speed factor in Study 3 was nearly identical to that in Study 2, and so it is not pictured; please see Figure 4). 

#### 5.2.1. Interruption Frequency

The aggregate interruption rate across all learners and interaction types was 2.165%. 

#### 5.2.2. Interruptions and COVID-19

**Figure 7:**
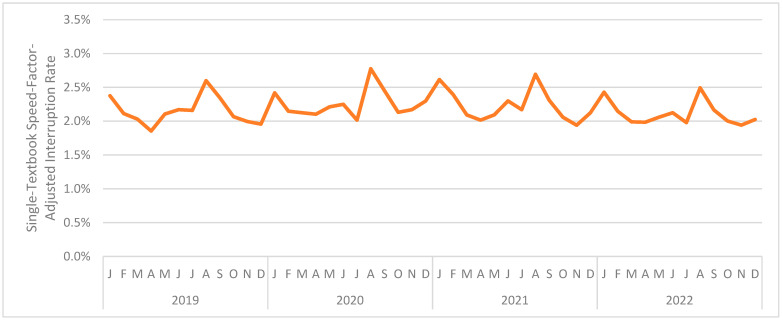
The monthly interruption rate for Study 3.

Figure 7 presents the interruption rate (combined across interaction types) by month. The (unweighted) mean monthly interruption rate was 2.187%. The month with the highest mean interruption rate was August 2020 at 2.775%. The month with the lowest mean interruption rate was April 2019 at 1.855%. As can be seen, and as was the case in Studies 1 and 2, the interruption rate was relatively consistent across the period under analysis.

Table 5 presents the interruption counts, interaction counts, and interruption rates for the time periods corresponding to our hypothesis. As before, the numeric differences in the interruption rates appear trivial. 

A test of equal proportions indicated that pre-COVID, peak COVID, and post-peak interruption rates were statistically significantly different: χ^2^(2, *N* = 96,688,318) = 287.03, *p* ≈ 0. Comparing pre-COVID to peak COVID yielded an effect size of *h* = −0.00003. Comparing pre-COVID to post-peak yielded an effect size of *h* = −0.00363. Comparing peak COVID to post-peak yielded an effect size of *h* = −0.00360. As in Studies 1 and 2, these differences are negligible.

Table 6 presents the interruption counts, interaction counts, and interruption rates for the seasonality-controlled time periods in our hypotheses. As in Table 5, the numeric differences in interruption rates appear trivial. 

A test of equal proportions again indicated that interruption rates in the four time periods were statistically significantly different: χ^2^(3, *N* = 14,484,123) = 467.42, *p* ≈ 0. Comparing 2020 to 2019 yielded an effect size of *h* = 0.01512. Comparing 2020 to 2021 yielded an effect size of *h* = 0.00697. Comparing 2020 to 2022 yielded an effect size of *h* = 0.00931. Textbook and edition usage variances appear unlikely to have obscured an impact of COVID-19 on interruption rate in Studies 1 and 2. 

## 6. Study 4 

In Studies 1–3, we defined an interruption as any interaction with an item longer than the mean + 2 SD for that type of interaction with that item (with adjustments in Studies 2 and 3 for individual differences). The two-standard-deviation threshold is popular but arbitrary (Leys et al. 2013). There is just as much justification to have chosen 2.5 or 3 (Miller 1991), or even 2.08 or 2.12. Furthermore, extreme outliers themselves increase both the mean and the standard deviation; the threshold incorporates the very aberrations it is intended to detect (e.g., Owuor et al. 2022).

Study 4 was designed to reduce both the arbitrary elements of outlier identification and the influence of those outliers on their detection threshold. Iglewicz and Hoaglin (1993) make the case that a *modified Z-score* can achieve both these objectives. A modified Z-score is based on the *median absolute deviation* (MAD), which in this case is the median of interactions’ absolute deviations from the median interaction duration. Leys et al. (2013) give the following formula:

$$\mathrm{MAD}=\mathrm{med}(|x_{i}-\mathrm{med}(x)|)$$

An item’s modified Z-score $M_{i}$ uses the MAD as the unit of measure for calculating an interaction’s “distance” from the median. Iglewicz and Hoaglin (1993) give the following formula:

$$M_{i}=\frac{0.6745(x_{i}-\mathrm{med}(x))}{\mathrm{MAD}}$$

Because the expected value of MAD in large data sets is 0.6745 median-equivalents of the standard deviation, including that coefficient in the numerator makes the expected value of $M_{i}$ equal to zero (Iglewicz and Hoaglin 1993). Simulations indicate that a threshold value of 3.5 for the modified Z-score is ideal for identifying outliers (Iglewicz and Hoaglin 1993). Compared to Studies 1–3, these data-driven constants reduce the subjectivity of outlier detection in Study 4, and the modified Z-score’s reliance on the MAD makes it far more robust than relying on the mean and standard deviation (Leys et al. 2013). 

### 6.1. Method

The research population and materials were the same as in Studies 1 and 2. 

### 6.2. Results and Discussion

**Figure 8:**
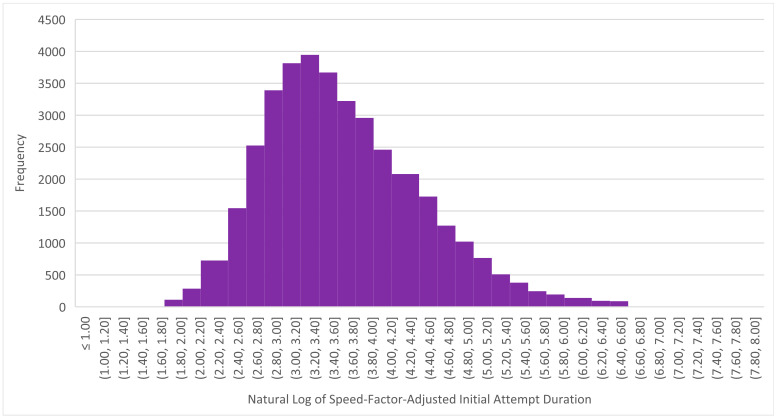
Distribution of the transformed durations of initial interactions with the example item from Figure 1.

The modified Z-score method is appropriate with data that are mildly skewed but less so with data that are highly skewed (Saleem et al. 2021). We therefore needed to reduce the strong skew in our data (exemplified in Figure 1 and Figure 2). We also needed to take the speed factor into account. We therefore first adjusted the duration of each interaction according to the learner’s speed factor by dividing the duration by the speed factor. This approach was isomorphic to the approach we took in Studies 1 and 2, where we multiplied the interruption threshold by the speed factor. We then took the natural log of the adjusted duration (a common transformation for skewed data; Osborne 2002). Figure 8 presents the transformed data for the item depicted in Figure 1; visual inspection indicates that it is indeed roughly normal and only mildly skewed. 

For each interaction, MADs were separately calculated for learners’ (a) initial attempts, (b) subsequent attempts, (c) initial reviews of corrective feedback, and (d) subsequent reviews of corrective feedback. Within each of these interaction types for each item, interruptions were defined as speed-factor-adjusted interactions whose natural log had a modified Z-score above 3.5 (Iglewicz and Hoaglin 1993). 

#### 6.2.1. Interruption Frequency

To measure the aggregate interruption rate across all learners and content, we divided the total number of interruptions by the total number of interactions, combined across interaction types. The result was an overall mean interruption rate of 1.214%.

Although we had no formal hypotheses about it, the overall interruption rate with the modified-Z-score method was lower than we had expected. Miller (1991) characterizes a mean + 2 SD threshold as liberal but only for normal distributions. Our (untransformed) data’s positive skew—the long right tail—moves that threshold further to the right, making it more conservative. Given MAD’s robustness (i.e., its resistance to outliers; Leys et al. 2013), we expected the modified Z-score would yield a lower threshold and, thus, a higher interruption rate than in Studies 1–3.

#### 6.2.2. Interruptions and COVID-19

**Figure 9:**
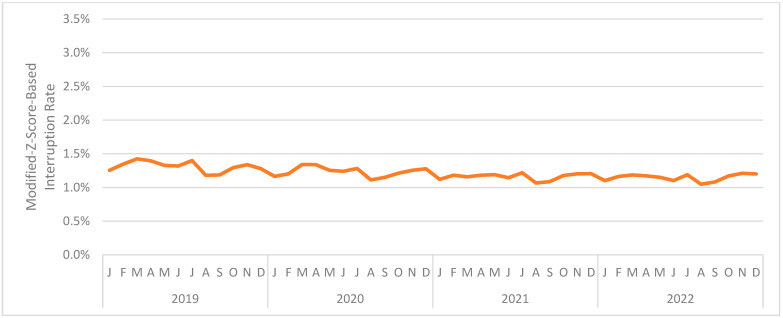
The monthly interruption rate for Study 4.

Figure 9 presents the interruption rate (combined across interaction types) by month. The (unweighted) mean monthly interruption rate was also 1.214%. The month with the highest mean interruption rate was March 2019 at 1.425%. The month with the lowest mean interruption rate was August 2022 at 1.049%. As before, the interruption rate was relatively consistent across the period under analysis.

Table 7 presents the interruption counts, interaction counts, and interruption rates for the time periods corresponding to our hypothesis. As with the mean + 2 SD method, the numeric differences in the interruption rates appear trivial. 

A test of equal proportions indicated that pre-COVID, peak COVID, and post-peak interruption rates were statistically significantly different: χ^2^(2, *N* = 995,687,507) = 30,559, *p* ≈ 0. Comparing pre-COVID to peak COVID yielded an effect size of *h* = −0.00175. Comparing pre-COVID to post-peak yielded an effect size of *h* = 0.01133. Comparing peak COVID to post-peak yielded an effect size of *h* = 0.01308. As in Studies 1 and 2, these differences are negligible.

Table 8 presents the interruption counts, interaction counts, and interruption rates for the seasonality-controlled time periods in our hypotheses. As in Table 7, the numeric differences in interruption rates appear trivial.

A test of equal proportions again indicated that interruption rates in the four time periods were statistically significantly different: χ^2^(3, *N* = 152,778,376) = 9213.5, *p* ≈ 0. Comparing 2020 to 2019 yielded an effect size of *h* = −0.00544. Comparing 2020 to 2021 yielded an effect size of *h* = 0.01153. Comparing 2020 to 2022 yielded an effect size of *h* = 0.01323. The true effect of lockdowns on interruptions in the present study is effectively zero. 

These findings are consistent with Studies 1, 2, and 3. Contrary to our hypothesis, COVID-19 did not meaningfully disrupt learning in the online educational platform used in the present evaluation. 

## 7. Study 5

In the present framework, interruptions are defined as interaction durations so long that they must be anomalous. In Studies 1–4, we used increasingly sophisticated methods to identify statistical duration-of-interruption outliers. 

On the other hand, there is subjectivity inherent to any claim that a datum is an outlier (Zimek and Filzmoser 2018). It is the act of declaring that an element should not be in a set that it is already in. As such, there is always the potential for error. Although we have attempted to rule out systematic influences on those errors, some unconsidered source of variance may yet have affected our conclusions. 

In Study 5, we leveraged the constraints of the educational software platform to identify extreme-duration interruptions with 100% certainty. In addition to this certainty, longer interruptions are disproportionately more damaging for learning and are therefore more important to detect (Monk et al. 2008).

### 7.1. Defining Long Interruptions

In most educational software platforms, learners log in and make progress during a digital *session*. After a period of inactivity during which a learner does not interact with the system, the session ends, and the learner is logged out. The duration of inactivity that results in a logout is pre-defined. In the digital platform used in the present investigation, the session timeout is triggered after 30 min of inactivity. 

For the present analysis, we defined a *long interruption* as the timeout of a digital session before a module was completed. (The completion took place in another digital session.) A single module could have multiple long interruptions; for example, a module that was completed over four sessions was said to have had three long interruptions.

This definition does not capture all interruptions. It does, however, accurately identify events that completely disengage a learner from the learning activity, such as taking a nap, going to the movies, or heading out on a camping trip. Critically, even if the learner was not interrupted by some other event, the timeout itself was an interruption because it logged the learner out. The learner had to log back in and resume interacting with the learning material to complete the module. As a result, we can be certain that every long interruption truly represents interrupted learning activity. 

### 7.2. Results and Discussion

#### 7.2.1. Analysis Note

In September 2019, a change was made to the software platform’s method for capturing data about the end of a session. Data before that time are unsuitable for comparison to data after that time. Thus, in Study 5, all data were from modules started and completed between October 2019 and December 2022, inclusive. 

#### 7.2.2. Long-Interruption Frequency

To measure the aggregate long-interruption rate across all learners and content, we divided the sum of long interruptions by the sum of interactions. The overall result was a mean long-interruption rate of 0.351%. In other words, long interruptions only occur approximately once every 285 interactions. 

Because long interruptions are only the subset of interruptions that exceed 30 min, this rate does not represent the overall rate of interruptions during online learning. It can, however, be used to evaluate the potential impact of COVID-19. 

#### 7.2.3. Interruptions and COVID-19

**Figure 10:**
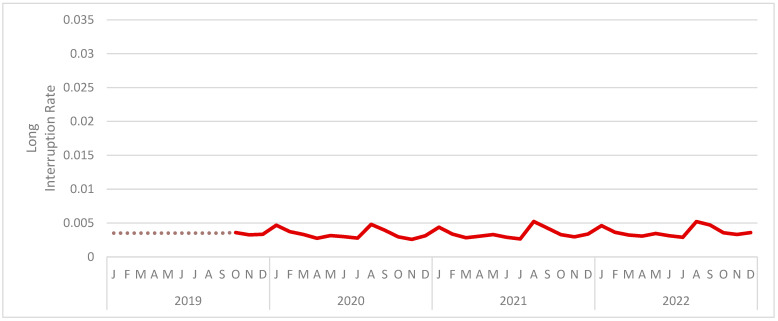
The monthly long-interruption rate for Study 5. Values from before October 2019 have been replaced with the average of all subsequent values (represented by a dotted line).

Figure 10 presents the long-interruption rate by month. The (unweighted) mean monthly long-interruption rate was also 0.351%. The month with the highest mean long-interruption rate was August 2021 at 0.524%. The month with the lowest mean long-interruption rate was November 2020 at 0.258%. As in all previous studies, the rate was relatively consistent across the period under analysis.

Table 9 presents the long-interruption counts, interaction counts, and long-interruption rates for the time periods corresponding to our hypothesis. As in all previous studies, the numeric differences across time periods appear trivial. 

A test of equal proportions indicated that pre-COVID, peak COVID, and post-peak interruption rates were statistically significantly different: χ^2^(2, *N* = 817,589,585) = 5723.6, *p* ≈ 0. Comparing pre-COVID to peak COVID yielded an effect size of *h* = 0.01399. Comparing pre-COVID to post-peak yielded an effect size of *h* = 0.00248. Comparing peak COVID to post-peak yielded an effect size of *h* = −0.01151. As in Study 1, these differences are negligible. 

Table 10 presents the long-interruption counts, interaction counts, and long-interruption rates for the seasonality-controlled time periods in our hypotheses. As in Table 9, the numeric differences across time periods appear trivial. 

A test of equal proportions indicated that interruption rates in the three time periods were statistically significantly different: χ^2^(2, *N* = 113,279,304) = 708.09, *p* ≈ 0. Comparing 2020 to 2021 yielded an effect size of *h* = −0.00456. Comparing 2020 to 2022 yielded an effect size of *h* = −0.00576. The true effect of lockdowns on interruptions in the present study is effectively zero.

## 8. General Discussion

Across five studies, we explored various ways of establishing the rate at which online learners were interrupted while using an adaptive educational platform. By employing a traditional mean + 2 SD threshold with adjustments based on learners’ speed, we found that approximately 2.855% of interactions were interrupted (Study 2). When the threshold was established using a learner-speed-adjusted modified Z-score—a more robust, objective estimator—the rate was 1.214% (Study 4). Software timeouts unambiguously capture long interruptions; they occurred at a rate of 0.351% (Study 5). 

These findings are complementary. That is, Studies 2 and 4 were designed to detect interruptions that occurred during a single session in the software platform. A learner might have gotten up to feed a pet or move laundry from a washer to a dryer, but they resumed the module before the 30 min software timeout. On the other hand, Study 5 captured interruptions that exceeded that timeout. A more complete picture of a learner’s interruption rate can therefore be obtained by combining these within-session and session-ending interruptions. Combining the long interruptions from Study 5 with the speed-adjusted mean + 2 SD approach from Study 2 gives an overall interruption rate of 3.206%. Combining long interruptions instead with the modified-Z-score-based approach from Study 4 gives an overall interruption rate of 1.565%. 

In all cases, COVID-19 and lockdowns did not appear to affect the interruption rate. The statistical significance in the differences between pre-COVID, peak COVID, and post-peak periods was a result of the massive sample size—with just over one billion interactions evaluated—but the magnitude of the effects was nearly indistinguishable from zero. 

It is important not to take these results as evidence that COVID-19 and lockdowns were anything less than catastrophically disruptive to everyday life and education; they were. Indeed, we remain surprised at the pattern of results given the breadth and magnitude of stressors that COVID-19 and lockdowns created. Future research could evaluate the interruption rate in other digital learning systems to determine whether the present platform’s immunity came from its being based on findings taken from the science of learning, the fact that it was already online pre-pandemic (and was therefore not emergency-interrupted; Hodges et al. 2020), or some other factor. 

### 8.1. Implications for Digital Education

Given the direct and indirect negative effects that interruptions can have on learning (e.g., Schneegass et al. 2022), it is important to detect and respond to them as early in a student’s educational experience as possible. We present straightforward approaches for doing so, given the limited data that may be available in a web-based educational software platform. Even the modified Z-score, although more complex than the mean-and-standard-deviation approach, can be readily calculated in common productivity software (e.g., Microsoft Excel or Google Sheets), making it appropriate for an instructor evaluating their students. The modified Z-score also minimizes subjectivity, giving an instructor firm ground on which to stand (i.e., to know and perhaps be able to convince their students that they are being evaluated in a fair and unbiased way).

An instructor who discovers a student is frequently interrupted might invite that student to office hours to discuss their study habits. A software platform that automatically detected these students could provide a report to the instructor to support such an invitation. The software platform might also be modified to provide metacognitive guidance in real time, helping the learner re-engage with the module. With enough data, learners might be directed toward improving their study environment or prioritizing educational activities at times of day when their learning is less prone to interruption.

### 8.2. Other Interruption-Detection Approaches 

The outlier-detection approaches we took were intended to have clear face validity, be straightforward to implement in commonly available software, and minimize the subjectivity on the part of the investigator. We considered but rejected several other approaches that, in our view, did not meet these criteria. One approach is to treat the distribution of duration data as being mostly generated by a process that represents engaged learning and then attempting to find the properties of that distribution, including the outliers (e.g., Motulsky and Brown 2006). An alternative, machine-learning approach is to treat the data as a mixture of two distributions—genuine interactions and interruptions—and then train a computer *classifier* to distinguish data that come from one distribution versus the other (e.g., Joachims 1998). A third approach is to use a clustering algorithm to determine which data points are most similar to one another; the main group contains the inliers, and the distant scatter represents the outliers (e.g., Campello et al. 2013). 

Each of these methods is difficult to implement and requires critical subjective steps, (e.g., Zimek and Filzmoser 2018), such as declaring a priori the family of an underlying distribution or manually setting a threshold (e.g., Tukey 1977). Other machine-learning approaches require training data, where a researcher has coded interactions as interrupted or not, and the computer uses this information to learn to distinguish normal from interrupted interactions (e.g., Mohri et al. 2012). Because the software platform in the present investigation is used remotely via web interface, there can be no human-coded interruption data, ruling out these *supervised learning* approaches. However, investigations in which more types of data are collected and coded (e.g., eye tracking; Faber et al. 2018) may benefit from machine-learning approaches to classifying interruptions (although caution is warranted to the extent that these methods become intrusive; (Calderwood et al. 2014). 

### 8.3. Future Research

#### 8.3.1. Gaming the System

Even without additional sources of data, the present findings invite several avenues of study. For example, future researchers might use modified versions of the methods described to evaluate outliers at the other end of the spectrum. The quick responses at the far left of Figure 1 and Figure 2 may have come from users who are “gaming the system” or otherwise attempting to rush to completion. Research by Baker et al. (2004) indicates that this type of behavior may be more strongly correlated with reduced learning than any other off-task behavior. And follow-up analyses on research by Murphy et al. (2022) indicate that almost half of all students rush through recorded video lectures even when they believe that rushing will impair learning. For many students, completion is more important than comprehension. Detecting and correcting this kind of behavior could have meaningful, positive effects on student outcomes. 

#### 8.3.2. The Impact of Interruption Rate

Directly evaluating the relationship between interruption rate and learning, perhaps in an experiment with a delayed post-test, could also be valuable. Interruptions have a wide variety of negative consequences for learning (e.g., Sitzmann et al. 2010), but there may be some interruption-rate threshold above which learning quality or efficiency meaningfully deteriorates. For students above this threshold, an instructor intervention soon after an assignment or a real-time intervention by the software itself could correct the course of their instruction. 

#### 8.3.3. Computer versus Classroom

Other future investigations could tackle a wide variety of issues. For example, although prior researchers have compared online learning to in-person instruction (e.g., Bernard et al. 2004; Koory 2003), it may be valuable to directly compare interruption rates when web-based modules are completed remotely versus in the classroom. A related line of investigation would be to compare interruptions in online modules completed by students at traditional, hybrid, and online-only universities—and in flipped versus traditional classrooms. Future researchers may also consider evaluating the relationship between demographics and distraction; there are likely substantial differences in the nature and prevalence of distractions for a sophomore at a four-year, in-person university versus a full-time employee who is completing online courses after putting three children to bed.

#### 8.3.4. Recalculating Speed Factor 

In the present study, we took individual student variation into account with the inclusion of the speed factor in Studies 2–4. Future researchers may wish to explore alternative ways to calculate the speed factor. For example, rather than dividing the sum of all learner durations by the sum of all mean durations, we could have used the (weighted) mean of the learner-to-group duration ratios. 

We could also have used the median rather than the mean, which would have made the speed factor itself less affected by outliers. Our preliminary analyses indicated that these approaches caused very few interactions to be recategorized as interrupted versus normal (or vice versa). Importantly, however, these approaches may yield different results with smaller data sets (such as one might find in a single instructor’s classroom).

#### 8.3.5. The Details of an Interruption

Future researchers may also be interested in delving into the details of an interruption. In the present study, for example, we cannot determine whether a 300 s interruption was a single 5 min event or ten separate 30 s disruptions. These two scenarios may have different implications for the quality of student learning. 

We also cannot determine whether an interaction was abnormally long because the learner was slowed by having their attention divided (e.g., by trying to ignore arguing neighbors) as opposed to having their attention completely disconnected from the educational software (e.g., by getting up and asking the neighbors to quiet down). Even if it does not rise to the level of a total interruption, “consistent interference” can drain attention and affect learning (Wang 2022). Future research might target the automatic identification of these partial distractions. 

Finally, whether partial or complete, it may also be valuable to investigate the differential impact of interruptions whose origin is internal (e.g., mind wandering; Smith et al. 2022) versus external (e.g., a ringing phone). Although only 11% of interruptions are of internal origin (Federman 2019), and this line of effort may be difficult in remote-learning systems with limited data (Draxler et al. 2021), these two types of disengagements may have different cognitive consequences; detecting and recovering from one may be more important than the other. 

## 9. Conclusions

Human intuition about learning is often misguided. Difficulty can be desirable in that it can lead to superior long-term learning. However, productive difficulty can feel undesirable, and learners often use this feeling to guide themselves away from optimal conditions of instruction (e.g., toward massing instead of spacing). Online learning platforms can sidestep these metacognitive errors, but are more vulnerable to interruptions, which can negatively impact learning. 

In remote-learning systems where direct observation of students is impossible, there are nevertheless valid statistical approaches for identifying when students are interrupted. We explored several methods for using duration-of-interaction data to find instances where learners spent an inordinate amount of time. Regardless of approach, interruptions during online learning were relatively infrequent, occurring during less than 3.5% of learners’ interactions. In short, students appear to be able to effectively navigate distractions to maintain engagement with a cognitive-science-based learning platform designed for online use. 

Contrary to our hypothesis, interruption rates in the present software platform were relatively consistent from 2019 through 2022. The massive disruptions to in-person education caused by the COVID-19 pandemic appeared to have limited spillover into an online platform designed from the start to educate remotely using robust learning principles. This result hints at what we might expect from the next massive disruption of student learning.

The interruption-detection methods we evaluated are straightforward enough for an instructor to implement using common productivity software (e.g., Microsoft Excel). The resulting information could allow an instructor to identify students who are frequently interrupted during their use of digital educational tools and who may therefore be struggling to learn due to external factors rather than a lack of effort or aptitude. Additionally, if the software platform could use these methods to detect an abundance of interruptions in real time, it could provide metacognitive guidance to help students better manage their learning environment before even a single lesson was completed. These approaches could enhance learners’ engagement, facilitate meaningful knowledge acquisition, and promote successful student outcomes in the environment of online education. 

## Figures and Tables

**Table 1:** The interruption data for the three periods corresponding to our hypothesis.

	Pre-COVID	Peak COVID	Post-Peak
Interruption count	6,107,869	830,424	13,129,965
Interaction count (N)	301,995,418	41,255,775	652,436,314
Interruption rate	2.023%	2.013%	2.012%

**Table 2:** The interruption data for the seasonality-controlled time periods corresponding to our hypothesis.

	April–May 2019	Peak COVID	April–May 2021	April–May 2022
Interruption count	745,691	830,424	732,269	641,015
Interaction count (N)	39,499,072	41,255,775	38,079,051	33,944,478
Interruption rate	1.888%	2.013%	1.923%	1.888%

**Table 3:** The interruption data for the three periods corresponding to our hypothesis.

	Pre-COVID	Peak COVID	Post-Peak
Interruption count	8,289,573	1,128,205	19,020,378
Interaction count (N)	301,995,418	41,255,775	652,436,314
Interruption rate	2.745%	2.735%	2.915%

**Table 4:** The interruption data for the seasonality-controlled time periods corresponding to our hypothesis.

	April–May 2019	Peak COVID	April–May 2021	April–May 2022
Interruption count	980,407	1,128,205	1,042,978	914,577
Interaction count (N)	39,499,072	41,255,775	38,079,051	33,944,478
Interruption rate	2.482%	2.735%	2.739%	2.694%

**Table 5:** The interruption data for the three periods corresponding to our hypothesis.

	Pre-COVID	Peak COVID	Post-Peak
Interruption count	636,550	81,864	1,375,571
Interaction count (N)	29,865,657	3,840,171	62,982,490
Interruption rate	2.131%	2.132%	2.184%

**Table 6:** The interruption data for the seasonality-controlled time periods corresponding to our hypothesis.

	April–May 2019	Peak COVID	April–May 2021	April–May 2022
Interruption count	80,077	81,864	67,116	63,348
Interaction count (N)	4,173,068	3,840,171	3,302,522	3,168,362
Interruption rate	1.919%	2.132%	2.032%	1.999%

**Table 7:** The interruption data for the three periods corresponding to our hypothesis.

	Pre-COVID	Peak COVID	Post-Peak
Interruption count	3,902,034	541,229	7,615,344
Interaction count (N)	301,995,418	41,255,775	652,436,314
Interruption rate	1.292%	1.312%	1.167%

**Table 8:** The interruption data for the seasonality-controlled time periods corresponding to our hypothesis.

	April–May 2019	Peak COVID	April–May 2021	April–May 2022
Interruption count	542,901	541,229	450,813	395,661
Interaction count (N)	39,499,072	41,255,775	38,079,051	33,944,478
Interruption rate	1.374%	1.312%	1.184%	1.166%

**Table 9:** The long-interruption data for the three periods corresponding to our hypothesis.

	Pre-COVID	Peak COVID	Post-Peak
Long-interruption count	454,676	118,497	2,297,449
Interaction count (N)	123,897,496	41,255,775	652,436,314
Long-interruption rate	0.367%	0.287%	0.352%

**Table 10:** The long-interruption data for the seasonality-controlled time periods corresponding to our hypothesis. Values from before October 2019 have been omitted; please see the Analysis Note.

	April–May 2019	Peak COVID	April–May 2021	April–May 2022
Long-interruption count	--	118,497	118,871	108,245
Interaction count (N)	--	41,255,775	38,079,051	33,944,478
Long-interruption rate	--	0.287%	0.312%	0.319%

## Data Availability

Restrictions apply to the availability of these data. Data were obtained from Amplifire, Inc., and are available on request from the corresponding author with permission from Amplifire, Inc.

## Notes

1 For an explanation on why these effects can be interpreted as medium or even large, please see Kraft (2020).

2 The difference versus the overall rate is due to the varying numbers of interactions per month.

3 The degrees of freedom in this test and the overall N do not match because March 2020 was excluded, as mentioned above.

4 Separate analyses were performed for each interaction type to further accommodate individual differences (e.g., a learner who answered quickly but read corrective feedback slowly, or whose initial attempts were slow but whose subsequent attempts were quick).
